# Decomposing the rural–urban gap in the prevalence of undiagnosed, untreated and under-treated hypertension among older adults in India

**DOI:** 10.1186/s12889-022-13664-1

**Published:** 2022-07-08

**Authors:** Bandita Boro, Shreya Banerjee

**Affiliations:** Centre for the Study of Regional Development, School of Social Sciences, Jawaharlal Nehru University, New Delhi, India

**Keywords:** Rural–urban gap, Hypertension, Older adults, Decomposition analysis, Health-seeking behavior

## Abstract

**Background:**

Although awareness and treatment rates of hypertension have significantly improved in recent years, the prevalence of undiagnosed and untreated hypertension remains a major public health concern for Indian policymakers. While the urban–rural variation in the prevalence, diagnosis, control, and treatment of hypertension is reasonably well-documented, the explanation behind such variation remains poorly understood given the dearth of studies conducted on exploring the determinants of the rural–urban gap in the prevalence of undiagnosed, untreated, and uncontrolled hypertension in India. In view of this research gap, our paper aims to decompose the inter-group differences between rural and urban areas in undiagnosed, untreated, and undertreated hypertension among older adults in India into the major contributing factors.

**Methods:**

Nationally representative data collected in the Longitudinal Ageing Study of India, Wave-1 (2017–18), was utilized for this study. Maximum-likelihood binary logistic-regression models were employed to capture the crude and adjusted associations between the place of residence and prevalence of undiagnosed, untreated, and undertreated hypertension. Fairlie’s decomposition technique was used to decompose the inter-group differences between rural and urban residents in the prevalence of undiagnosed, untreated, and undertreated hypertension among the older population in India, into the major contributing factors, in order to explore the pathways through which these differences manifest.

**Results:**

The overall prevalence rates of undiagnosed, untreated, and undertreated hypertension among older adults were 42.3%, 6%, and 18.7%, respectively. However, the prevalence of undiagnosed and untreated hypertension was higher in rural areas, by 12.4 and 1.7 percentage-points, respectively, while undertreated hypertension was more prevalent in the urban areas (by 7.2 percentage-points). The decomposition analysis explained roughly 41% and 34% of the urban advantage over rural areas in the case of undiagnosed and untreated hypertension, while it explained 51% of the urban disadvantage in respect of undertreated hypertension. The rural–urban differentials in education and comorbidities accounted for the majority of the explained rural disadvantage in the prevalence of undiagnosed hypertension, explaining 13.51% and 13.27% of the gap, respectively. The regional factor was found to be the major driver behind urban advantage in the prevalence of untreated hypertension, contributing 37.47% to the overall gap. In the case of undertreated hypertension, education, comorbidities, and tobacco consumption were the major contributors to the urban–rural inequality, which accounted for 12.3%, 10.6%, and 9.8% of the gap, respectively.

**Conclusion:**

Socio-economic and lifestyle factors seemed to contribute significantly to the urban–rural gap in undiagnosed, untreated and undertreated hypertension in India among older adults. There is an urgent need of creating awareness programmes for the early identification of hypertensive cases and regular treatment, particularly in under-serviced rural India. Interventions should be made targeting specific population groups to tackle inequality in healthcare utilization.

## Background

Non-Communicable Diseases (NCDs) such as heart diseases, stroke, diabetes, cancer and chronic respiratory diseases are the leading causes for morbidity and mortality worldwide, with three-fourth of deaths occurring in the low and middle-income countries after the age of 60 [1]. Among them, hypertension is the leading cause of mortality [2] and is ranked third as the risk factor of healthy years of life lost due to morbidity or pre-mature death (disability-adjusted life) [3]. Hypertension is a major risk factor for cardiovascular diseases (CVD), particularly ischemic heart disease and stroke [4]. In the recent years, the burden of hypertension has increased substantially in the low-income and middle-income countries and in South Asia it is the third most important risk factor for disease burden [5]. More than 35% of the adult population are affected by hypertension in the Asian region thereby becoming a serious public health concern [6].The burden of hypertension has been projected to multiply by 2025 in India and China [7].

Although awareness and treatment rates of hypertension have significantly improved in recent years, prevalence of undiagnosed and untreated hypertension still remains a major public health issue plaguing the developing societies [8]. The low- and middle-income countries have a higher rate of undiagnosed, uncontrolled and untreated hypertension than in the developed countries [1]. Lack of knowledge, detection and treatment of hypertension contribute to higher risk of stroke, younger age of onset and larger proportion of intracerebral haemorrhage in lower-income countries [9]

Previous studies have documented the prevalence of undetected, untreated or uncontrolled hypertension to be highly associated with lower socio-economic status such as living in rural areas, lower educational attainment and low income level [10–13]. The difference in prevalence of hypertension between urban and rural regions worldwide varies in both magnitude and direction [14]. A number of studies have documented a higher prevalence of hypertension and its associated risk factors in urban areas compared to the rural areas [15–17]. While some studies have found the awareness, treatment and control rates to be lower in urban areas than rural areas [16, 18, 19], a few other studies have found evidence suggesting otherwise, i.e. prevalence rates of awareness, treatment and control of hypertension are much lower in rural areas as compared to their urban counterparts [15, 20, 21].

There is a substantial body of research depicting a significant urban–rural difference in overall health care utilization among older adults in India disfavouring the rural residents owing to the poor health-care provisions in terms of quality and outreach in rural India [22, 23]. Additionally, studies addressing the issue of health-seeking behaviour specifically for hypertension have found that the prevalence of self-reported hypertension is much lower than the actual prevalence of hypertension when cross-verified with measurement of blood pressure during survey [24–26]. For example, a recent study using cross-sectional data found the self-reported prevalence of hypertension to be only 5.5% compared to the actual (measured) prevalence of hypertension at 26.3% in India thereby highlighting the presence of a wide care deficit [27]. Another study estimated the prevalence of undiagnosed hypertension among women aged 15–49 years to be 18.63% at the national level and 17.09% and 21.73% in rural and urban areas, respectively, clearly indicating an urban disadvantage [28].

While the rural–urban variation in the prevalence, diagnosis, control and treatment of hypertension is reasonably well documented, the explanation behind such variation is not well attempted and there is a paucity of studies conducted on exploring the determinants of the rural–urban gap in the prevalence of undiagnosed, untreated and uncontrolled hypertension in India. In a country like India, with a larger socio-economically disadvantaged population living mostly in rural areas with limited health care facility, the actual burden of undiagnosed, untreated or uncontrolled hypertension remains poorly understood. In view of this research gap, our paper aims to examine the association between place of residence and prevalence of undiagnosed, untreated and undertreated hypertension among older adults aged 45 and above in India, on the one hand and to decompose the inter-group differences between rural and urban areas, in the same, into the major contributing factors, on the other hand.

## Materials and methods

### Data source

**Fig. 1:**
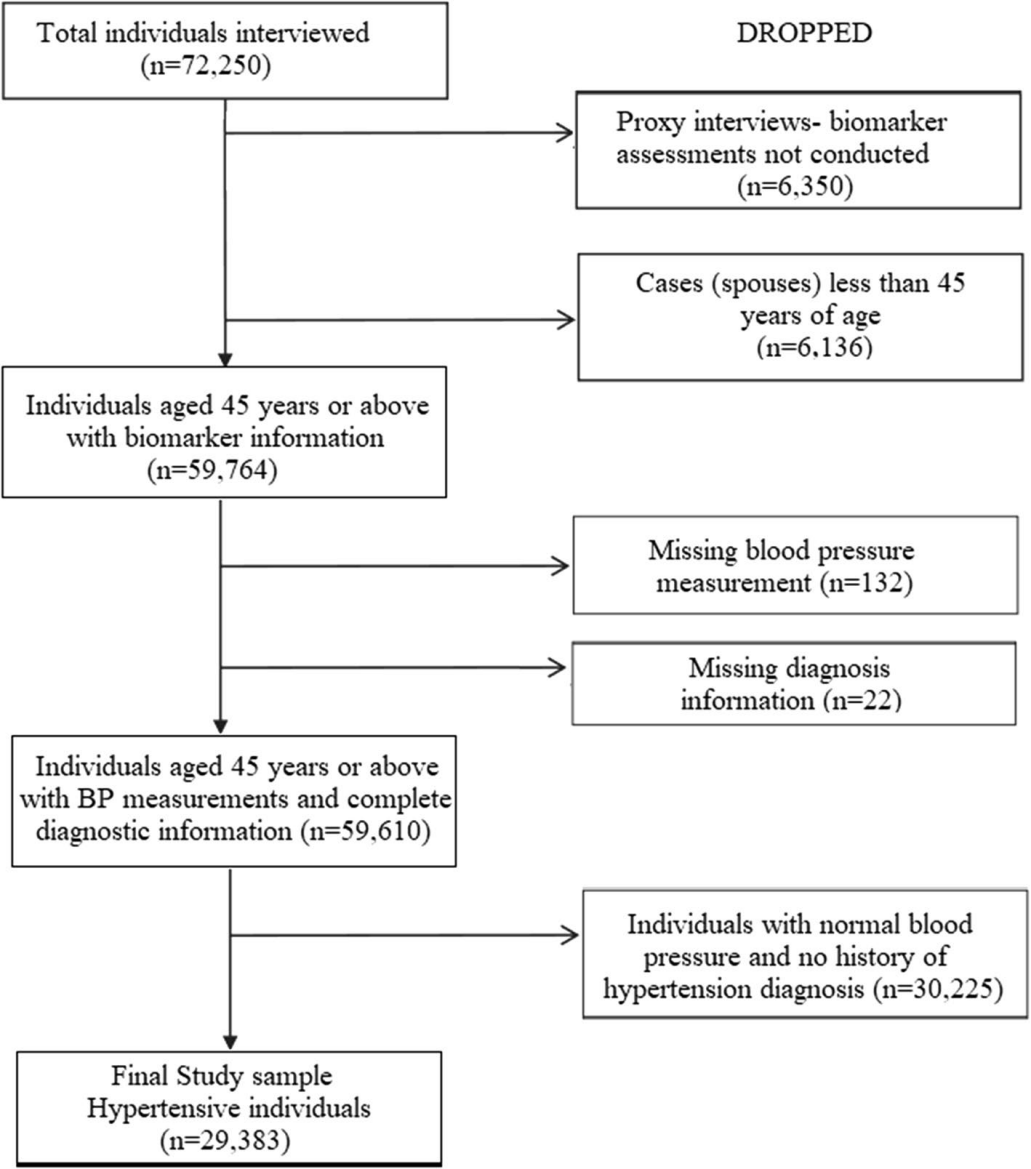
Schematic representation of inclusion/ exclusion criteria of study participants

The analysis has been done drawing evidence from the data collected through the Longitudinal Ageing Study of India (Wave-1), 2017–18, a nationally representative large-scale sample survey. Adopting a multi-stage stratified area probability cluster sampling design,^1^ the LASI interviewed 72,250 older adults aged 45 and above^2^ (including their spouses irrespective of age) across all states and union territories of India, except Sikkim, covering 42,949 households. The survey collected data on the health, economic and social well-being of older adults in India. In addition to self-reported data on morbidity, the LASI also conducted internationally validated direct health examinations for a more accurate and objective measure of health and disease-burden. The full range of biological markers included in the LASI comprises physiological, performance-based, anthropometric and dried blood spot based molecular measurements. However, in case the selected respondent had severe cognitive or physical impairment, a proxy interview was done, in which case, biomarker assessments were not conducted. For the present analysis, only the respondents aged 45 years or above whose biomarker tests were conducted were considered. Moreover, cases where the blood pressure measurements or diagnosis history were missing were also dropped, leaving a gross sample of 59,610 individuals (39,007 rural and 20,603 urban dwellers). Of these, only the hypertensive individuals (29,383; 17,668 rural and 11,715 urban residents) were retained for the analyses pertaining to unmet need of healthcare. Figure 1 provides a schematic representation of the process of selection of participants for the present study.

### Outcome Variables

**Fig. 2:**
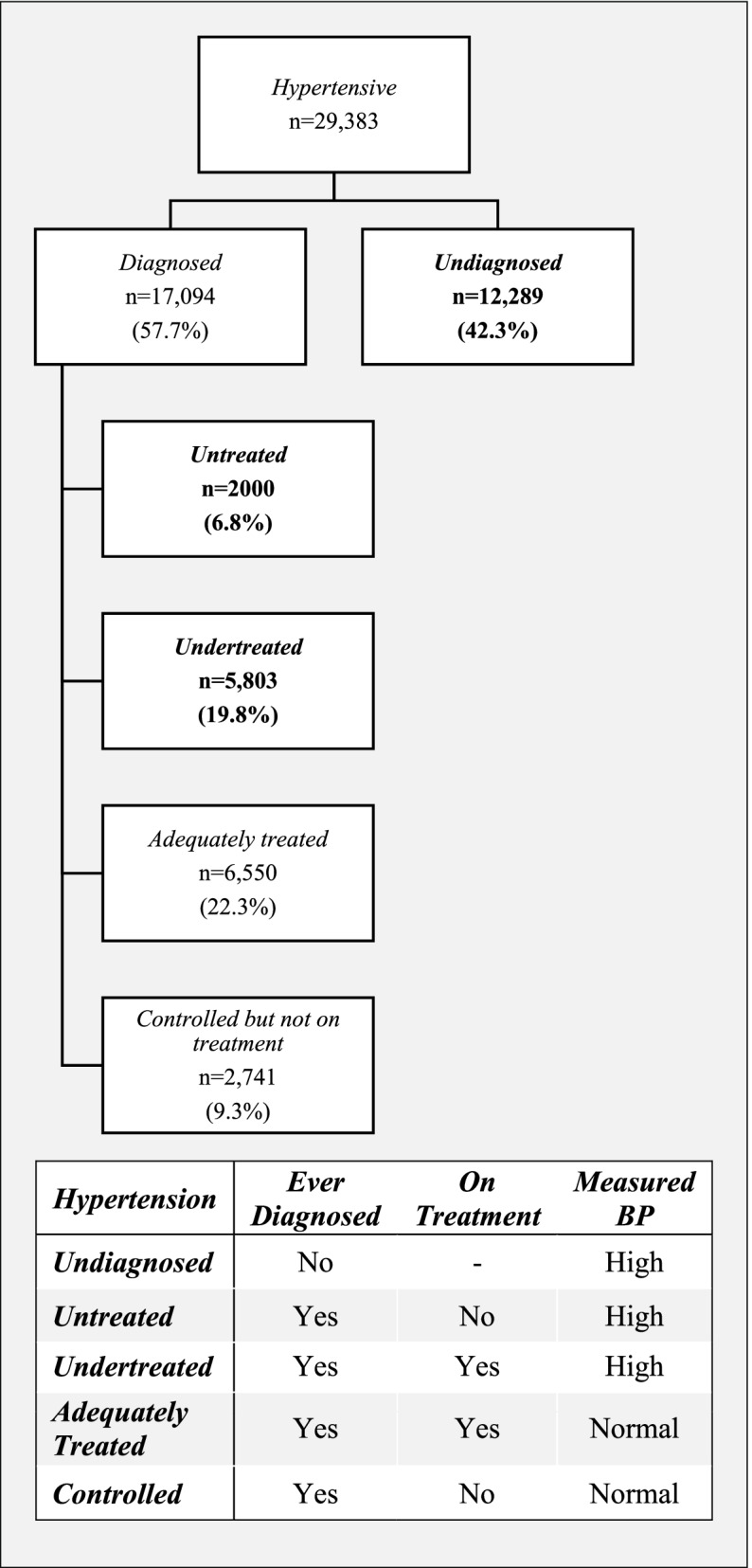
The continuum of care for hypertension: unmet need of healthcare. Note: The weighted prevalence of unmet need of healthcare is presented as percentages in parentheses. Each prevalence rate is calculated keeping the total number of hypertensive individuals (29,383) as the base, i.e., the base was not restricted to the number of individuals reaching the preceding stage of the continuum

The LASI, in its module on ‘diseases and health conditions’, collected self-reported information on the history of diagnosis of and treatment for several chronic health conditions including hypertension. The questions were framed as: ‘has any health professional ever diagnosed you with hypertension or high blood pressure? (yes/ no)’, ‘in order to control your blood pressure or hypertension, are you currently taking any medication? (yes/ no)’, etc. Additionally, blood pressure measurements were also recorded by the surveyors using an ‘Omron HEM 7121’ BP monitor, adopting internationally comparable protocols. Three measurements of blood pressure were taken, with one-minute gap between each of the measurements.^3^ The mean of the last two measurements were used to calculate blood pressure. A raised blood pressure refers to a mean systolic blood pressure ≥ 140 mmHg and/or mean diastolic blood pressure ≥ 90 mmHg, as per the standard classification protocol recommended by the World Health Organisation (WHO). In the present study, an individual was considered hypertensive if they either had a raised blood pressure (measured) or if they reported to have ever been diagnosed with hypertension by a health professional, or both. Based on the self-reported history of diagnosis and treatment as well as the objective measurement of blood pressure, the outcome variables were defined as follows (Fig. 2).

***Undiagnosed hypertension:*** If the individual reported to have never been diagnosed with hypertension by a health professional but their measured mean systolic blood pressure was ≥ 140 mmHg or diastolic blood pressure was ≥ 90 mmHg or both.

***Untreated hypertension:*** If the individual reported to have been diagnosed with hypertension by a health professional and their measured mean systolic blood pressure was ≥ 140 mmHg or diastolic blood pressure was ≥ 90 mmHg or both but are currently not receiving any treatment.

***Undertreated hypertension:*** If the individual reported to have been diagnosed with hypertension by a health professional and are currently receiving treatment but their measured mean systolic blood pressure was ≥ 140 mmHg or diastolic blood pressure was ≥ 90 mmHg or both.

### Predictor variables

Place of residence has been established as an important axis of inequality in access to and utilisation of healthcare, in general and geriatric care, in particular, disfavouring the rural residents over their urban counterparts [23, 31]. The main predictor of our model was thus constituted of place of residence, categorised as rural and urban.

Additionally, a set of covariates pertaining to five broad domains were also included in our models. These domains included demographic factors, socio-economic factors, institutional-support factor, geographical factor and health-risk and behavioural factors.

The demographic factors comprised sex (male and female), age (grouped as 45–59 years and 60 years or above), marital status (currently married and others including never married/ divorced/ separated/ widowed), religion (Hindus, Muslims and other minority religious groups like Sikhs, Christians etc.), and social groups ((Scheduled Castes (SC), Scheduled Tribes (ST), Other Backward Classes (OBC) and others). Age and age-squared were included as a continuous variables in the multivariate analyses to model the effect of age more accurately, which may have a non-linear relationship with the outcomes.

The socio-economic factors included economic status (Monthly Per-capita Consumption Expenditure based quintiles), education (not literate, primary or below, secondary, and higher secondary or above) and work status (never worked, currently not working and currently working). Health insurance coverage (covered and not covered), irrespective of type of coverage scheme and benefits was included as an institutional-support factor. While region (north, central, east, northeast, west and south) was included as a geographical factor.

Finally, a set of health risk and behavioural factors known to be associated with hypertension prevalence and chances of diagnosis were also identified. These included comorbidities^4^ (none and at least one), tobacco consumption^5^ (never consumed in any form, currently not consuming in any form, smokes tobacco, uses smokeless tobacco and uses both smokable and smokeless tobacco), Body Mass Index- weight in kilograms divided by square of height in metres (underweight if below 18.5, normal if in the range 18.5–24.9 and overweight if 25 or above) and physical activity (inactive if performs below 150 min of moderate-intensity activities daily, moderately active if engages in 150–300 min of daily physical activities of moderate intensity and highly active if performs more than 300 min of such activities daily, as per WHO guidelines^6^

### Statistical analyses

Descriptive statistics were calculated to understand the distribution of the study sample as a whole as well as rural–urban wise, by select background characteristics. Bivariate percentage distribution was calculated to estimate the differentials in the prevalence of undiagnosed, untreated and undertreated hypertension by predictor variables. The results were tested for statistically significant independence using Pearson’s Chi-squared test statistic.

Maximum likelihood binary logistic regression models were employed to capture the crude and the adjusted association between place of residence and prevalence of undiagnosed, untreated and undertreated hypertension. The multivariate model on adjusted association between unmet need of healthcare and residence controlled for all the covariates comprising the demographic, socio-economic, institutional support, regional and health risk and behavioral factors. The results are presented as crude and adjusted odds ratios with 95% confidence intervals.

Finally, Fairlie’s decomposition technique was used to decompose the inter-group differences between rural and urban residents, in the prevalence of undiagnosed, untreated and undertreated hypertension among the older population in India, into the major contributing factors [32, 33]. The Fairlie’s decomposition technique is a non-linear approximation of the Blinder-Oaxaca decomposition method [34, 35]. The decomposition analysis was undertaken using the pooled estimated coefficients of both the two groups. The *fairlie* command [36] in STATA version 16 was used with randomised ordering of the variables and 5000 decomposition replications. The sampling weights were applied in the analyses to account for the complex sample design and non-response as per the LASI (2017–18).

## Results

### Profile of the study participants

Table 1 shows the profile of the study participants included in our study. More than two-third (70%) of the older adults belonged to the rural areas. Besides, of the total study participants, 54% were females, 74% were currently married, 83% were Hindus, 46% belonged to Other Backward Classes (OBCs), and 42% belonged to the bottom two wealth quintiles while 37% belonged to the two upper-most wealth quintiles. Participants were equally distributed over the two age categories of 45–59 years and 60 years or above (50% each). Majority of the older adults (74%) were either not literate or had an educational attainment of primary school or below, and 44% were currently employed in paid work. An overwhelming majority (80%) of the respondents were not covered by any health insurance scheme. Most of the participants belonged to the southern (24%) or eastern region (23%).

**Table 1:** Rural–urban differential in select characteristics of the study sample, LASI (2017-2018)

		**Total**		**Rural**		**Urban**	
**Background characteristics**		Freq	%	Freq	%	Freq	%
**Place of Residence**	Rural	39,007	69.9				
	Urban	20,603	30.1				
**Sex**	Male	27,593	45.9	18,238	46.7	9355	43.9
	Female	32,017	54.1	20,769	53.3	11,248	56.1
**Age group**	45–59 years	31,129	49.8	20,050	51.4	11,079	53.8
	60 years & above	28,481	50.2	18,957	48.6	9524	46.2
**Marital Status**	Currently married	44,881	74.2	29,566	75.0	15,315	72.4
	Others	14,729	25.8	9441	25.0	5288	27.7
**Religion**	Muslim	7085	11.0	3764	9.6	3321	14.2
	Hindu	43,726	82.5	29,071	84.0	14,655	79.2
	Others	8799	6.4	6172	6.4	2627	6.6
**Social Group**	SC	10,036	19.4	7315	22.3	2721	12.7
	ST	10,460	8.6	8089	10.8	2371	3.3
	OBC	22,488	45.7	14,660	44.1	7828	49.3
	Others	16,626	26.4	8943	22.8	7683	34.7
**Economic Status**	Poorest	11,791	21.1	7614	20.6	4177	22.2
	Poorer	12,021	21.3	7820	21.9	4201	19.9
	Middle	12,039	20.4	7910	21.0	4129	19.1
	Richer	12,014	19.6	7879	19.5	4135	20.0
	Richest	11,745	17.6	7784	17.1	3961	18.7
**Education**	Not literate	29,730	53.5	23,315	62.8	6415	32.0
	Primary or below	13,201	20.6	8235	20.0	4966	22.0
	Secondary	10,990	16.2	5539	12.5	5451	24.7
	Higher secondary or above	5689	9.7	1918	4.7	3771	21.3
**Work Status**	Never worked	16,330	26.1	9187	22.2	7143	35.4
	Currently not working	17,094	29.5	11,236	29.9	5858	28.7
	Currently working	26,186	44.3	18,584	48.0	7602	35.9
**Health Insurance**	Covered	13,794	20.4	9643	21.0	4151	18.9
	Not covered	45,816	79.7	29,364	79.0	16,452	81.1
**Region**	North	10,976	12.7	6881	12.6	4095	12.8
	Central	8181	21.0	6378	23.7	1803	14.7
	East	10,735	23.7	8092	27.7	2643	14.5
	Northeast	7726	3.4	5773	4.0	1953	2.1
	West	7846	15.8	4086	13.1	3760	22.2
	South	14,146	23.4	7797	18.9	6349	33.7
**Comorbidity**	None	30,983	51.5	21,607	53.9	9376	45.7
	At least one	28,627	48.6	17,400	46.1	11,227	54.3
**Physical Activity**	Inactive	38,339	62.9	23,646	60.3	14,693	68.8
	Moderately active	8609	14.6	5507	13.7	3102	16.8
	Highly active	12,662	22.5	9854	26.0	2808	14.4
**Tobacco Consumption**	Never consumed	37,603	62.3	22,665	57.2	14,938	74.1
	Currently not consuming any	3291	4.9	2154	5.1	1137	4.4
	Smokes only	7138	11.8	5350	13.5	8.68	7.7
	Uses smokeless tobacco only	10,478	19.3	7961	22.1	12.22	13.0
	Both smokable and smokeless	1100	1.8	877	2.1	223	0.9
**Body Mass Index**	Normal	31,443	52.0	21,766	54.8	9677	45.5
	Underweight	10,949	21.2	9170	26.2	1779	9.6
	Overweight	17,218	26.8	8071	19.1	9147	44.8
**Hypertension**	No	30,227	53.0	21,339	57.3	8888	43.1
	Yes	29,383	47.0	17,668	42.7	11,715	56.9
**TOTAL**		59,610	100.0	39,007	69.9	20,603	30.1

The percentages (%) are weighted

Source: Authors’ own calculations from Longitudinal Ageing Study in India, 2017–18 (LASI-Wave I)

Overall, 47% of the respondents were found to be hypertensive. The urban dwellers had a higher prevalence of hypertension than their rural counterparts by 14 percentage-points (43% rural; 57% urban). With respect to health risk and behavioural factors, 49% of the older persons had at least one comorbidity in addition to hypertension, 63% were physically inactive, 62% reported to have never consumed tobacco in any form while 32% currently use tobacco in either smokable or smokeless forms or both. In terms of BMI, 21% were underweight while 27% were overweight.

Urban areas observed a higher share of Muslims, adults with at least one comorbidity in addition to hypertension, those who never consumed tobacco of any type, those belonging to the two-richest wealth quintiles and adults found physically inactive by 4.6, 8.2, 16.9, 2.3 and 8.5 percentage points, respectively. On the other hand, rural areas had a higher share of adults aged 60 years or above, Scheduled Tribes, older adults who were not literate, currently working, and those with normal BMI by 2.4, 7.5, 30.8. 12.1 and 9.3 percentage points, respectively. Besides, urban areas were more concentrated in the southern and western region (55.9%) while rural areas were mostly located in the eastern and central region (51.4%).

### Rural–urban differential in the prevalence of unmet-need of healthcare for hypertension

Table 2 presents the rural–urban differences in the prevalence of undiagnosed, untreated and undertreated hypertension, all of which represent varying degrees of unmet need of healthcare for hypertension. The overall prevalence rates of undiagnosed, untreated and undertreated hypertension were 42.3%, 6% and 18.7%, respectively. However, the prevalence rates of undiagnosed and untreated hypertension were higher in rural areas, by 12.4 and 1.7 percentage points, respectively, while undertreated hypertension was more prevalent in the urban areas (by 7.2 percentage points).

**Table 2:** Rural–urban differential in prevalence of undiagnosed, untreated and undertreated hypertension among older adults by select background characteristics in India (2017–18)

		**Undiagnosed Hypertension**				**Untreated Hypertension**				**Undertreated Hypertension**			
**Background characteristics**		Total	Rural	Urban	R-U	Total	Rural	Urban	R-U	Total	Rural	Urban	R-U
**Sex**	Male	48.5	52.4	41.7	10.7	6.4 ^Ϯ^	6.8 ^Ϯ^	5.6 ^Ϯ^	1.2	16.7	14.0	21.4	-7.4
	Female	37.5	42.6	29.0	13.6	5.7	6.5	4.4	2.1	20.3	17.7	24.6	-6.9
**Age group**	45–59 years	46.0	48.6	41.9	6.7	5.8	6.2 ^Ϯ^	5.1	1.1	15.2	13.3	18.1	-4.8
	60 years & above	39.5	45.5	28.2	17.4	6.2	6.9	4.8	2.2	21.3	18.0	27.6	-9.6
**Marital Status**	Currently married	43.7	47.6	36.9	10.8	6.0	6.5	5.0 ^Ϯ^	1.5	17.2	15.0	21.0	-6.0
	Others	39.0	44.8	28.6	16.2	6.1	7.0	4.6	2.4	22.2	18.7	28.5	-9.8
**Religion**	Muslim	37.4	40.7	33.8 ^Ϯ^	6.9	6.1	7.0	5.2 ^Ϯ^	1.8	21.2	19.3	23.4	-4.1
	Hindu	43.2	47.9	34.7	13.2	5.9	6.5	4.9	1.6	18.0	15.3	22.9	-7.6
	Others	40.1	43.2	32.7	10.5	7.0	8.1	4.5	3.6	22.6	20.6	27.6	-7.0
**Social Group**	SC	44.8	46.5	39.2	7.3	7.6	8.4	5.0	3.4	16.5	15.4	20.4	-5.1
	ST	61.2	65.0	37.9	27.2	6.9	6.4	9.8	-3.4	10.7	9.5	18.2	-8.8
	OBC	42.2	45.5	37.1	8.4	5.3	5.9	4.3	1.6	18.8	16.4	22.6	-6.2
	Others	36.1	42.0	29.0	13.0	6.0	6.6	5.3	1.2	21.9	18.9	25.5	-6.6
**Economic Status**	Poorest	50.9	57.7	40.4	17.3	7.3	7.1 ^Ϯ^	7.6	-0.5	14.7	11.3	19.8	-8.5
	Poorer	45.1	50.4	34.7	15.7	7.0	7.6	5.6	2.0	17.3	14.0	23.6	-9.6
	Middle	43.9	47.2	37.7	9.4	5.9	6.8	4.3	2.5	18.5	17.0	21.3	-4.3
	Richer	37.0	40.6	30.9	9.7	5.5	6.6	3.5	3.1	22.1	17.7	29.7	-12.0
	Richest	34.9	38.7	28.4	10.4	4.5	5.1	3.6	1.5	20.8	20.2	21.8	-1.6
**Education**	Not literate	45.6	48.4	35.9 ^Ϯ^	12.5	6.3 ^Ϯ^	6.3	5.9	0.4	16.9	15.2	22.6 ^Ϯ^	-7.4
	Primary or below	40.5	43.9	35.2	8.7	5.6	6.5	4.0	2.5	19.2	16.5	23.5	-7.0
	Secondary	36.1	44.7	28.8	16.0	5.9	7.6	4.6	3.0	23.4	19.6	26.7	-7.1
	Higher secondary or above	40.1	44.2	38.3	5.9	6.0	8.7	4.8	3.9	18.9	17.1	19.7	-2.6
**Work Status**	Never worked	32.1	37.2	26.5	10.7	5.2	6.5	3.9 ^Ϯ^	2.6	23.1	20.0	26.4	-6.4
	Currently not working	38.3	42.3	30.1	12.2	6.9	7.4	5.9	1.5	21.5	19.1	26.2	-7.0
	Currently working	53.7	56.1	48.1	8.0	5.9	6.2	5.2	0.9	12.9	11.3	16.6	-5.3
**Health Insurance**	Covered	42.1 ^Ϯ^	46.5 ^Ϯ^	33.4 ^Ϯ^	13.1	5.8 ^Ϯ^	6.1 ^Ϯ^	5.1 ^Ϯ^	1.0	19.4 ^Ϯ^	17.9 ^Ϯ^	22.3 ^Ϯ^	-4.4
	Not covered	42.3	46.9	34.7	12.2	6.1	6.8	4.9	1.9	18.5	15.6	23.5	-7.9
**Region**	North	33.9	36.7	28.3	8.4	8.6	9.2	7.6	1.6	20.3	18.5	23.9	-5.4
	Central	49.2	54.4	36.8	17.6	6.0	5.5	7.3	-1.8	12.4	9.3	19.8	-10.5
	East	41.9	45.9	28.5	17.4	7.5	8.2	5.4	2.8	18.6	16.1	26.9	-10.9
	Northeast	41.6	43.9	32.7	11.2	10.4	11.3	6.6	4.7	20.6	18.8	27.1	-8.3
	West	45.6	51.9	38.4	13.5	5.1	5.6	4.6	1.1	17.8	14.1	21.9	-7.8
	South	40.3	45.0	35.6	9.4	3.4	3.7	3.0	0.7	22.6	21.6	23.6	-2.0
**Comorbidity**	None	59.2	61.8	53.7	8.1	5.4	5.8	4.6 ^Ϯ^	1.2	12.0	10.7	14.6	-3.9
	At least one	29.1	33.7	21.9	11.8	6.5	7.4	5.1	2.3	24.0	20.8	28.9	-8.1
**Physical Activity**	Inactive	39.3	43.7	32.2	11.6	5.9 ^Ϯ^	6.4 ^Ϯ^	5.1 ^Ϯ^	1.3	20.2	18.2	23.4	-5.2
	Moderately active	41.0	47.0	32.2	14.9	6.0	7.4	3.8	3.6	22.2	16.5	30.5	-14.0
	Highly active	54.1	55.6	49.5	6.1	6.6	6.9	5.4	1.5	10.7	9.6	13.9	-4.2
**Tobacco Consumption**	Never consumed	38.6	43.1	32.4	10.8	5.5	6.3	4.4	1.9	20.7	18.3	24.0	-5.8
	Currently not consuming any	37.8	42.3	28.6	13.7	7.7	9.2	4.6	4.6	22.3	17.9	31.3	-13.5
	Smokes only	52.4	53.0	50.7	2.3	7.1	6.9	7.9	-1.0	12.3	10.5	17.6	-7.1
	Uses smokeless tobacco only	50.4	54.2	39.2	15.0	6.9	7.1	6.2	0.9	14.7	13.2	19.0	-5.8
	Both smokable and smokeless	55.7	58.1	47.1	10.9	5.1	3.8	9.6	-5.8	8.9	7.2	14.9	-7.7
**Body Mass Index**	Normal	45.5	49.0	38.0	11.0	6.2	6.5	5.5	1.0	16.8	14.9	20.9	-6.0
	Underweight	52.6	54.0	45.1	8.9	6.8	7.0	6.0	1.0	9.7	8.9	14.0	-5.1
	Overweight	33.5	37.1	30.2	6.9	5.4	6.7	4.3	2.5	25.1	23.8	26.3	-2.5
**TOTAL**		42.3	46.8	34.4	12.4	6.0	6.7	4.9	1.7	18.7	16.1	23.3	-7.2

R Rural, U Urban; R-U percentage- point differences

All *p*-values for chi squared test statistic were below 0.05 except those marked.^Ϯ^

Source: Authors’ own calculations from Longitudinal Ageing Study in India, 2017–18 (LASI-Wave I)

Undiagnosed hypertension was more prevalent among the males, those aged between 45 and 59 years, currently married, Hindus, STs, poorest, not literate, currently working, without any comorbidities, highly physically active, use tobacco in both smokable and smokeless forms, underweight, and those located in the central region. The prevalence of undiagnosed hypertension was higher in case of rural areas across all sub-categories compared to urban areas. However, the rural–urban differential was the most pronounced in case of STs (by 27 percentage points), followed by central and eastern region, 60 year and above age-group and the poorest wealth quintile by 17.6, 17.4, 17.4 and 17.3 percentage points respectively.

Untreated hypertension had a higher prevalence in case of those aged 60 years or above, other minority religious groups, SCs, poorest wealth quintile, retired (currently not working), western region, have at least one comorbidity other than hypertension, have quit tobacco consumption (currently not consuming), and underweight. Untreated hypertension was more prevalent in rural areas compared to the urban for all sub-groups except in cases of STs, poorest, central region, and adults who are currently using tobacco. The rural–urban gap (disfavouring the rural), was observed to be the widest in case of those located in the northeastern region, who have quit tobacco use, and those with educational attainment of higher secondary or above, by 4.7, 4.6 and 3.9 percentage points, respectively.

Prevalence of undertreated hypertension was higher among older adults with the following characteristics: females, aged 60 years or above, currently not married, belonging to other minority religious groups, other social groups, richer wealth quintile, with at most secondary school education, never worked, located in the southern region, have at least one comorbidity, are moderately active, have quit tobacco use, and were overweight. Undertreated hypertension was consistently more prevalent in urban areas across all sub-categories. The rural–urban differential was the widest among those who were moderately active, have quit tobacco use, richer wealth quintile, and located in the eastern and central regions, by 14, 13.5, 12, 10.9, and 10.5 percentage points.

### Association between place of residence and unmet need of healthcare for hypertension

The crude and adjusted odds ratios computed through logistic regression to examine the association between place of residence and the prevalence of undiagnosed, untreated and undertreated hypertension have been presented in Table 3. In the crude model, the odds of an individual’s hypertension remaining undiagnosed was 68% higher in rural areas than the urban areas, while the odds of a diagnosed hypertension remaining untreated was 38% higher in rural areas. However, after adjusting for a range of covariates, the magnitude of the differentials shrunk while the direction remained unchanged, i.e., it continued to be in favour of the urban dwellers. In case of undertreated hypertension, the likelihood was lower in the rural areas by 37% in the crude analysis. In the adjusted model, however, the likelihood of inadequate treatment of hypertension was lower by only 15% in the rural areas compared to the urban.

**Table 3:** Crude and adjusted association between place of residence and prevalence of undiagnosed, untreated and undertreated hypertension among older adults in India (2017–18)

Predictors		Undiagnosed Hypertension		Untreated Hypertension		Undertreated Hypertension	
		COR	AOR	COR	AOR	COR	AOR
**Place of Residence**	Urban ®						
	Rural	1.68*** (1.44—1.95)	1.37*** (1.22—1.53)	1.38*** (1.16—1.63)	1.27** (1.07—1.51)	0.63*** (0.54—0.75)	0.85** (0.74—0.98)
**Sex**	Male ®						
	Female		0.70*** (0.6—0.81)		0.92 (0.75—1.13)		0.96 (0.81—1.12)
**Age**			0.96* (0.92—1.00)		1.02 (0.95—1.09)		1.08** (1.02—1.14)
**Age squared**			1.00 (0.99—1.00)		0.99 (0.99—1.00)		0.99** (0.99—1.00)
**Marital Status**	Others ®						
	Currently married		1.05 (0.93—1.18)		0.92 (0.76—1.11)		0.82** (0.69—0.98)
**Religion**	Muslim ®						
	Hindu		1.16* (0.98—1.36)		0.94 (0.75—1.17)		0.84* (0.7—1.00)
	Others		1.16 (0.94—1.42)		0.99 (0.72—1.35)		1.02 (0.8—1.3)
**Social Group**	SC ®						
	ST		1.64*** (1.39—1.94)		0.82 (0.61—1.11)		0.7** (0.55—0.89)
	OBC		1.07 (0.94—1.22)		0.83 (0.65—1.05)		0.93 (0.79—1.09)
	Others		1.04 (0.9—1.19)		0.78** (0.6—1.00)		1.04 (0.88—1.24)
**Economic Status**	Poorest ®						
	Poorer		0.86** (0.74—0.99)		0.89 (0.69—1.13)		1.14 (0.95—1.37)
	Middle		0.84** (0.71—1.00)		0.77** (0.61—0.97)		1.2* (1.00—1.46)
	Richer		0.7*** (0.6—0.82)		0.68** (0.53—0.88)		1.32** (1.08—1.62)
	Richest		0.68*** (0.57—0.81)		0.56*** (0.44—0.72)		1.14 (0.9—1.44)
**Education**	Not literate ®						
	Primary or below		0.81*** (0.71—0.92)		0.98 (0.78—1.22)		1.07 (0.91—1.25)
	Secondary		0.75*** (0.64—0.88)		1.13 (0.86—1.48)		1.19 (0.89—1.58)
	Higher secondary or above		0.88 (0.63—1.22)		1.35** (1.00—1.81)		0.95 (0.73—1.24)
**Work Status**	Not working ®						
	Currently working		1.36*** (1.21—1.53)		0.9 (0.74—1.09)		0.78*** (0.67—0.91)
**Health Insurance**	Not covered ®						
	Covered		0.95 (0.84—1.07)		0.96 (0.81—1.15)		1.07 (0.92—1.24)
**Region**	North ®						
	Central		1.41*** (1.22—1.63)		0.64*** (0.5—0.81)		0.77** (0.64—0.92)
	East		1.17** (1.03—1.34)		0.77** (0.63—0.94)		1.15* (0.98—1.34)
	Northeast		0.87* (0.74—1.01)		1.17 (0.93—1.49)		1.58*** (1.31—1.89)
	West		1.57*** (1.36—1.81)		0.56*** (0.42—0.75)		0.93 (0.79—1.1)
	South		1.48*** (1.26—1.73)		0.38*** (0.3—0.48)		1.11 (0.93—1.33)
**Comorbidity**	None ®						
	At least one		0.33*** (0.3—0.37)		1.28** (1.09—1.51)		1.83*** (1.62—2.07)
**Physical Activity**	Inactive ®						
	Moderately active		1.07 (0.92—1.25)		1.09 (0.85—1.39)		1.21 (0.93—1.57)
	Highly active		1.18** (1.01—1.38)		1.21*(0.98—1.5)		0.72*** (0.6—0.86)
**Tobacco Consumption**	Never consumed ®						
	Currently not consuming any		0.92 (0.73—1.15)		1.24 (0.86—1.81)		1.06 (0.78—1.44)
	Smokes only		1.24** (1.05—1.47)		1.16 (0.89—1.5)		0.71** (0.58—0.88)
	Uses smokeless tobacco only		1.28*** (1.13—1.44)		1.08 (0.88—1.33)		0.78** (0.67—0.91)
	Both smokable and smokeless		1.31* (0.97—1.77)		0.76 (0.44—1.3)		0.52** (0.32—0.87)
**Body Mass Index**	Normal ®						
	Underweight		1.14** (1.00—1.3)		0.99 (0.78—1.26)		0.56*** (0.47—0.67)
	Overweight		0.75*** (0.67—0.84)		1.04 (0.88—1.23)		1.53*** (1.33—1.75)

®Reference category; COR: Crude Odds Ratio, AOR: Adjusted Odds Ratio

^***^ *p* < 0.001 ** *p* < 0.05 and * *p* < 0.1

Source: Authors’ own calculations from Longitudinal Ageing Study in India, 2017–18 (LASI-Wave I)

Female older adults were 30% less likely to have their hypertension undiagnosed than the males. With increasing age, the likelihood of undiagnosed hypertension tends to decline. Hindus were 16% more likely to have undiagnosed hypertension than the Muslims. STs had the highest likelihood of undiagnosed hypertension among all the social groups. With upward movement in the socio-economic gradient (wealth and education), the likelihood of a missing diagnosis of hypertension decreases. Those currently engaged in paid work were 36% more likely to have an undiagnosed hypertension than those who never worked or have retired. With respect to region, northeast region had the lowest likelihood of undiagnosed hypertension while the western region had the highest. Moreover, those who were overweight or had some comorbidities were less likely to have their hypertension undiagnosed than those who have a normal BMI or do not have a comorbidity. Also, those who are highly physically active and use some forms of tobacco are more likely to have an undiagnosed hypertension than those who are physically inactive or have never consumed or quit tobacco.

In case of untreated hypertension, the statistically significant determinants were social group, economic status, education, region, comorbidity and physical activity. ‘Other’ social groups were 22% less likely than SCs to have an untreated hypertension; upper wealth quintiles were less likely to not receive treatment for a diagnosed hypertension than the lower quintiles. Those with education of higher secondary or above were more likely to have untreated hypertension than those not literate; southern region had the lowest likelihood of untreated hypertension among all the regions; those with at least one comorbidity and those highly active were more likely to not be on treatment for a diagnosed hypertension than those with no comorbidity and physically inactive adults.

Further, the adjusted model revealed a monotonic increasing function of undertreated hypertension by an individual’s age until a turning point is reached, after which the function starts to decrease. Currently married adults were less likely to have an undertreated hypertension than others, while Hindus were less likely than Muslims, currently working less likely than others, and STs less likely than SCs. The upper wealth quintiles showed a higher likelihood of undertreated hypertension than the poor. Education was not a statistically significant determinant of undertreated hypertension. Central region had the lowest likelihood of undertreatment among all the regions. Highly active adults and those consuming some forms of tobacco were less likely to receive undertreatment than physically inactive adults and non-users of tobacco. Overweight adults were 53% more likely to have an uncontrolled hypertension despite being on treatment than those with normal BMI. Also, adults with at least one comorbidity were also more likely (by 83%) to have an undertreated hypertension than those with none.

### Determinants of rural–urban differential in unmet need of healthcare for hypertension

Table 4 presents the results of the decomposition analysis conducted to delineate the relative contribution of the rural–urban differential in each of the covariates to the rural–urban differences in the prevalence of undiagnosed, untreated and undertreated hypertension among the older adults in India. The set of covariates included in our model explains roughly 41% and 34% of the urban advantage over rural areas in case of undiagnosed and untreated hypertension, while it explains 51% of the urban disadvantage in respect of undertreated hypertension. In case of undiagnosed hypertension, education, comorbidities, tobacco use, social group, work status, BMI, religion and physical activity were the major contributors in expanding the rural–urban gap, while the regional factor, economic status and age offset some of the urban advantage. In case of untreated hypertension, the rural–urban differential in the regional factor was the stand-alone, major, statistically significant determinant of the explained urban advantage over its rural counterpart. The rural–urban differential in comorbidities was observed to offset a part of the rural disadvantage in untreated hypertension. With respect to undertreated hypertension, the factors that induced the urban disadvantage were education, comorbidity, tobacco consumption, work status, BMI and religion, in order of their relative contribution. Age and economic status, however, contributed to contract the gap to a limited extent.

**Table 4:** Decomposition of the rural–urban gap in prevalence of undiagnosed, untreated and undertreated hypertension among older adults in India (2017–18)

	**Undiagnosed Hypertension**		**Untreated Hypertension**		**Undertreated Hypertension**	
**Urban**	0.3441		0.0492		0.2325	
**Rural**	0.4678		0.0665		0.161	
**Difference (U-R)**	-0.1236		-0.0174		0.0715	
	**Coefficients**	**%**	**Coefficients**	**%**	**Coefficients**	**%**
**Sex**	0.0002	-0.16	-0.00007	0.4	0.0001	0.14
**Age**	0.0012**	-0.97	-0.00005	0.29	-0.0011**	-1.54
**Marital Status**	0.0001	-0.08	0.00005	-0.29	-0.0002	-0.28
**Religion**	-0.0021*	1.7	-0.00004	0.23	0.0014*	1.96
**Social Group**	-0.0074***	5.99	0.00013	-0.75	0.0032	4.48
**Economic Status**	0.0017***	-1.38	0.00022	-1.26	-0.0006**	-0.84
**Education**	-0.0167***	13.51	-0.00011	0.63	0.0088*	12.31
**Work Status**	-0.0069***	5.58	0.00045	-2.59	0.005***	6.99
**Health Insurance**	0.0002	-0.16	0.00005	-0.29	-0.0002	-0.28
**Comorbidity**	-0.0164***	13.27	0.00087**	-5.00	0.0076***	10.63
**Physical Activity**	-0.0018*	1.46	-0.00049	2.82	0.0004	0.56
**Tobacco Consumption**	-0.0081***	6.55	-0.00039	2.24	0.007***	9.79
**Body Mass Index**	-0.0032**	2.59	0.00006	-0.34	0.0031**	4.34
**Region**	0.009***	-7.28	-0.00652***	37.47	0.0017	2.38
***Explained***	***-0.0502***	***40.61***	***-0.00584***	***33.56***	***0.0362***	***50.63***
***Unexplained***	***-0.0734***	***59.39***	***-0.01156***	***66.44***	***0.0353***	***49.37***

^***^ *p* < 0.001 ** *p* < 0.05 and * *p* < 0.1

Source: Authors’ own calculations from Longitudinal Ageing Study in India, 2017–18 (LASI-Wave I)

## Discussion

The study showed the rural–urban inequality in the prevalence of undiagnosed, untreated and undertreated hypertension among the older population in India over the age of 45 years. The overall prevalence rates of undiagnosed, untreated and undertreated hypertension were found to be 42.3%, 6% and 18.7%, respectively. Concurrent with the findings of other studies with a similar objective, our study indicated the presence of inequalities in the prevalence of undiagnosed and untreated hypertension disfavoring the rural areas, by 12.4 and 1.7 percentage points, respectively [15, 16, 18, 19]. This can be explained by the fact that the availability of healthcare facilities are better in urban areas than in rural areas [31, 37, 38]. Moreover, inaccessibility due to poor transport and communication, absenteeism of health staff, more dependence on traditional medicines are factors known to be responsible for low utilization of health care services in the rural areas [39, 40].

Socio-economic and lifestyle factors seemed to contribute significantly to the urban–rural gap in undiagnosed, untreated and undertreated hypertension in India among older adults. This is similar to the findings of previous studies in India where undiagnosed and untreated hypertension was higher among those individuals living in rural areas and with lower educational attainment [41–43]. We found the prevalence of undiagnosed hypertension to be lower in higher educated participants. This may be a reflection of the fact that educated people in addition to having better knowledge of healthy lifestyles, also have relatively more affordability and accessibility to medical services compared to the lower educated participants [21]. Moreover, people belonging to Scheduled Tribes are associated with lower awareness and poorer treatment seeking behaviour which results in delayed diagnosis or no diagnosis at all [40, 44]. The higher share of illiterate and STs in the rural population were, therefore, seen to be major contributors of the rural disadvantage in the prevalence of undiagnosed hypertension.

Interestingly, our study found a negative association between the existence of at least one chronic co-morbidity and undiagnosed hypertension. This may be due to incidental diagnosis of hypertension when the individuals present themselves at a health facility seeking treatment for a different disease or health condition. The rural–urban differential in comorbidities was therefore a significant contributor of the rural–urban differential in the prevalence of undiagnosed hypertension. For similar reasons (of incidental diagnosis), abnormal BMI, associated with higher morbidity risk was a significant, albeit minor contributor of the rural–urban differential in undiagnosed hypertension. Obesity/ overweight along with sedentary lifestyle increases the risk of hypertension along with other adverse health conditions [45–47].

Moreover, currently working older adults were associated with a higher risk of undiagnosed hypertension. The proportion of currently working older adults were found to be higher in the rural areas, which added to the rural disadvantage in undiagnosed hypertension. Lack of pension and social security in the informal jobs and in farming compels the older adults to continue working at a lower wage beyond the statutory age of retirement. Also, the the share of population working in the white collar jobs having more access to health services and ability to afford treatment is higher in the urban areas [41, 42]. Tobacco consumption was also significantly associated with undiagnosed hypertension [48]. The rural India had a higher proportion of older adults currently consuming tobacco in some form which aggravated the rural–urban gap in undiagnosed hypertension disfavouring the rural residents.

Studies have depicted that there is a lack of awareness due to low accessibility of health care services among the poorer economic sections in both rural and urban areas. A recent study in India depicted that diagnosis and treatment rates of hypertension were lower not only for poorer and less educated individuals but also among the lower age groups and rural dwellers [43], resonating with the findings of our study. Since the urban population has a lower share of older age group population and higher share of poor population, the economic status and age played a role in offsetting the urban advantage in prevalence of undiagnosed hypertension.

Further, the regression results showed that adults with at least one comorbidity had higher odds of having their hypertension untreated compared to those with no comorbidities. The urban population has a higher share of older adults with comorbidities than the rural areas, which explains why the rural–urban differential in comorbidities was observed to offset a part of the rural disadvantage in untreated hypertension. Further, studies need to be carried to gain insights as to why untreated rates are almost similar in both rural and urban in spite of the fact that urban areas have more access to health facilities.

A systematic analysis found that only 11% and 20% of rural and urban Indians, respectively, had their BP under control [49]. However, contrastingly, in our study, the uncontrolled or under-treated hypertension was found to be higher in the urban areas by 7.2 percentage points. Our study corroborates with the findings of previous studies that wealth and education are important determinants in both control and treatment of hypertension [50]. Studies have found that sedentary lifestyle and unhealthy eating habits are higher in urban areas leading to obesity and eventually results in uncontrolled hypertension despite treatment [51, 52]. A study using NFHS 4 data has found that the likelihood of having uncontrolled hypertension was relatively higher for tobacco-users at the lowest wealth quintile and with no education, highlighting a source of health disparities in India [51]. The rural–urban differential in age distribution and economic status, however, contributed to contract the urban disadvantage in prevalence of undertreated hypertension to a limited extent. Previous literature shows that age is most strongly related to systolic blood pressure and isolated systolic hypertension and mostly older adults had a higher prevalence of undertreated (uncontrolled) hypertension [53, 54].

The present study highlighted a significant urban–rural disparity in the diagnosis, treatment, and control of hypertension in India. The findings showed that rural hypertensive adults had lower diagnosis and treatment rates than their urban counterparts did, while undertreated hypertension was higher for urban older adults. The higher rates of undiagnosed, untreated and undertreated hypertension among the poor, less educated and people living in the rural areas call for an urgent need for an accessible and affordable primary health-care system in the country. The key strength of this study is in the use of a recently released nationally representative sample. In addition, we have supplemented self-reported hypertension with measured BP, which rules out the self-reporting bias. However, there are limitations in this study too. The cross-sectional nature of the data limits the causal understanding of the associations studied. Another limitation is that there was no data on adherence to prescribed treatment, and so it is not possible to examine the influence of non-adherence on uncontrolled hypertension. Most clinical guidelines recommend the practice of confirming a high blood pressure at a later time through a second BP measurement for accurate diagnosis of hypertension [55]. However, in the survey data used, BP was measured only at a single occasion (albeit three successive readings were taken with a gap of one minute each). This may have resulted in an overestimation/ underestimation of hypertension to some extent. Furthermore, due to data limitations, white-coat hypertension and masked hypertension, conditions in which a patient’s blood pressure readings are inaccurate due to the nature of settings in which BP measurements are taken [56], couldn’t be accounted for. Despite these limitations, the present study made a reasonable contribution to the understanding of the contributing factors of the rural–urban gap in undiagnosed, untreated and under-treated hypertension among the older adults in India.

## Conclusion

The significant high burden of undiagnosed, untreated and undertreated cases of hypertension among the older adults suggests for an urgent need of creating awareness programmes for early identification of cases and regular treatment, particularly in the under-serviced rural India. Socio-economic conditions are important factors in contributing to the urban–rural disparities and hence there should be interventions targeting specific populations based on education, wealth and age. The health care providers should address behavioural risk factors, particularly unhealthy diet, tobacco consumption and physical inactivity in order to prevent hypertension. The health care facilities in the rural areas should be improved in terms of diagnosis and screening facilities and easy access to low-cost or free antihypertensive medications. Special attention should be given to those with existing co-morbid conditions since it gives rise to further complications.

## Data Availability

The data (Longitudinal Ageing Survey of India, Wave-1) used for the present analysis is freely available for academic researchers and can be requested from here: https://www.iipsindia.ac.in/content/data-request

## Abbreviations

NCDs: Non-Communicable Diseases
CVDs: Cardio-vascular diseases
WHO: World Health Organisation
LASI: Longitudinal Ageing Survey of India
NFHS: National Family Health Survey
BP: Blood Pressure
SCs: Scheduled Castes
STs: Scheduled Tribes
OBC: Other Backward Class
BMI: Body Mass Index
COR: Crude Odds Ratio
AOR: Adjusted Odds Ratio

## Footnotes

1 Within each of the Indian States and Union Territories (except Sikkim), the LASI Wave-1 enrolled subjects through a three-stage sampling selection procedure in rural areas and a four-stage sampling selection procedure in urban areas. In each state and UT, the first stage involved selecting Primary Sampling Units (PSUs) constituting sub-districts, i.e., Tehsils or Talukas. In the second stage, villages in rural areas and wards in urban areas were selected within each PSU, previously selected in the first stage. In case of rural areas, the third and final stage involved selecting households from each of the selected villages. While in urban areas, an additional stage was adopted whereby one Census Enumeration Block (CEB) was randomly selected in each urban ward followed by selection of households from each of these CEBs [29].

2 While the onset of non-communicable chronic diseases, in most of the developed countries, typically occurs at the age of 55 years or above, in India, the onset has been found to occur a decade earlier, at age of 45 years or older [30]. Hence, cut-off age is important to be set at 45 years to study ageing and health transition from prime adult ages in the Indian context.

3 The BP measurements were taken on the left arm. In case the participant had a rash, a cast, edema (swelling) in the left arm, open sores or wounds, or a significant bruise where the blood pressure cuff was to be in contact, BP measurement was taken on the right arm. The following script was used by the surveyor to explain the procedure to the participant: “I would like to measure your blood pressure and pulse using this monitor and cuff which I will secure around your left arm. I would like to take three blood pressure measures. I will ask you to relax and remain seated and quiet, with legs uncrossed and feet flat on the floor, during the measurements. First, I will place the cuff on your left arm. Once the cuff is placed appropriately on your arm and we are ready to begin, I will ask you to lay your arm on a flat surface, palm facing up, so that the center of your upper arm is at the same height as your heart. I will then press the start button. The cuff will inflate and deflate automatically. It will squeeze your arm a bit, but won’t hurt. After we have completed all three measures, I will give you your results” [29].

4 In LASI, information was collected on several self-reported (diagnosed) chronic health conditions. Respondents were asked: ‘has any health professional ever diagnosed you with the following chronic conditions or diseases?’ The chronic conditions included hypertension, diabetes, cancer or a malignant tumour, chronic lung diseases, chronic heart diseases, stroke, bone/joint diseases, neurological or psychiatric diseases, and high cholesterol, in addition to other chronic conditions such as thyroid, skin, chronic gastrointestinal, and organ-related diseases. Comorbidity is defined as a condition whereby the participant reported to have been ever diagnosed (by a health professional) with at least one of these chronic conditions in addition to hypertension.

5 In LASI, information was collected on various domains of health behaviour and health risk factors including tobacco use, a primary risk factor of chronic cardiovascular diseases. Tobacco consumption occurs in various forms, broadly comprising two categories: smoked and smokeless. Smoked tobacco involves burning tobacco products (cigarette, bidi, cigar, hookah, cheroot) and inhaling the smoke, whereas smokeless tobacco involves consuming tobacco in forms other than smoking like chewing tobacco, gutka, pan masala, etc. that is widely used across India. In LASI, information was collected on ever and current use of tobacco- both smokable and smokeless tobacco use. Based on these three questions: “have you ever smoked tobacco or used smokeless tobacco? (yes/ no); do you currently smoke any tobacco products? (yes/ no); and do you currently consume any smokeless tobacco products? (yes/no/)”, we constructed five categories of tobacco consumption as follows: 1) never consumed tobacco in any form, 2) currently not consuming tobacco in any form, i.e., ever used tobacco in some form but now has quit all, 3) currently smokes tobacco only, 4) currently uses smokeless tobacco only, and 5) currently uses both smokable and smokeless tobacco.

6 World Health Organisation’s global recommendations on measuring physical activity: https://www.who.int/news-room/fact-sheets/detail/physical-activity
